# Shape Estimation of Soft Manipulator Using Stretchable Sensor

**DOI:** 10.34133/2021/9843894

**Published:** 2021-04-21

**Authors:** Jinho So, Uikyum Kim, Yong Bum Kim, Dong-Yeop Seok, Sang Yul Yang, Kihyeon Kim, Jae Hyeong Park, Seong Tak Hwang, Young Jin Gong, Hyouk Ryeol Choi

**Affiliations:** ^1^ Mechatronics R&D Center, Samsung Electronics, Republic of Korea; ^2^ Korea Institute of Machinery & Materials, Daejeon, Republic of Korea; ^3^ AIDIN ROBOTICS Inc., SuwonRepublic of Korea; ^4^ School of Mechanical Engineering, Sungkyunkwan University, Republic of Korea

## Abstract

The soft robot manipulator is attracting attention in the surgical fields with its intrinsic softness, lightness in its weight, and safety toward the human organ. However, it cannot be used widely because of its difficulty of control. To control a soft robot manipulator accurately, shape sensing is essential. This paper presents a method of estimating the shape of a soft robot manipulator by using a skin-type stretchable sensor composed of a multiwalled carbon nanotube (MWCNT) and silicone (p7670). The sensor can be easily fabricated and applied by simply attaching it to the surface of the soft manipulator. In its fabrication, MWCNT is sprayed on a teflon sheet, and liquid-state silicone is poured on it. After curing, we turn it over and cover it with another silicone layer. The sensor is fabricated with a sandwich structure to decrease the hysteresis of the sensor. After calibration and determining the relationship between the resistance of the sensor and the strain, three sensors are attached at 120° intervals. Using the obtained data, the curvature of the manipulator is calculated, and the entire shape is reconstructed. To validate its accuracy, the estimated shape is compared with the camera data. We experiment with three, six, and nine sensors attached, and the result of the error of shape estimation is compared. As a result, the minimum tip position error is approximately 8.9 mm, which corresponded to 4.45% of the total length of the manipulator when using nine sensors.

## 1. Introduction

Natural orifice transluminal endoscopic surgery (NOTES) is a new surgical method that has been studied in recent years. In this type of surgery, surgical tools such as forceps and scissors can be inserted through the mouth, anus, nose, vagina, and urethra [1–3]. Compared with minimally invasive surgery (MIS), NOTES involves less pain, less fear, and faster recovery time, because it is incisionless. Since it is difficult to access a surgical target using a traditional rigid manipulator through a tortuous anatomical path, a continuum manipulator that can provide sufficient workspace and is compliant in a confined space is essential to use NOTES. Numerous studies have been conducted to make continuum manipulators available for NOTES [4–8].

Recently, the soft continuum manipulator has received attention for its applications in surgery. Generally, there is a risk of puncturing a healthy organ during robotic surgery. Some studies have installed force torque sensors in the end effector to prevent excessive force on organs [9, 10]. However, it is inefficient to install sensors in many parts of the robot. However, a soft manipulator’s inherent compliance ensures safety when a collision with the human body occurs. Various studies have implemented the soft manipulator for safe robotic surgery. The STIFF-FLOP project is a prominent study on a soft manipulator applied in NOTES. It was bioinspired by the arm of the octopus and can reach the surgical target location in a confined environment and increase safety. Cianchetti et al. [11] made a single-module manipulator. It facilitates an omnidirectional bend and elongation using a flexible fluidic actuator. Ranzani et al. [12] extended it to a double module. Each module can be controlled independently, and workspace and stiffening capabilities are characterized. Stilli et al. [13] suggested a shrinkable soft manipulator that can elongate the longitudinal axis over a wide range. These studies are being conducted regularly in the STIFF-FLOP project. However, despite the advantages of the soft manipulator, it cannot be widely used due to the difficulty of control.

For an accurate control continuum manipulator, shape estimation is an essential technique. Roesthuis et al. [14] demonstrated the shape estimation of a continuum manipulator that greatly improved the trajectory tracking performance. A Fiber Bragg Grating (FBG) sensor is generally used for the shape estimation of a rigid continuum manipulator [14–16]. It is an optical sensor and is therefore biocompatible and immune from magnetic fields and electrical interference. However, intrinsic stiffness limits the allowable strain to approximately 1% [15]. This means that the FBG sensor should be located near the central axis. In this case, the space efficiency will be decreased. Additionally, to transfer strain from the continuum manipulator to the sensor, high stiffness of the continuum robot is required. This means that the FBG sensor is difficult to adjust in a soft manipulator [17]. One of the alternatives is to attach a strain gauge to the soft manipulator to measure displacement and estimate deformation. However, strain gauze, which is a limit of less than 10 percent, is limited in its use. At least 15% deformation is allowed to measure 90° or more.

Softening the sensor is a new alternative to the shape estimation of a soft manipulator. Recently, stretchable strain sensors have attracted attention in various applications. Their intrinsic flexibility and elasticity allow them to be mounted on human skin or a robot surface. This does not disturb the human’s or robot’s movement and is suitable for use in sensing biosignals, movement, wearable electronic devices, etc. [18–21].

A stretchable strain sensor is fabricated using diverse materials and methods. Amjadi et al. fabricated a sensor using a nanocomposite of silver nanowire (AgNW) and PDMS. They fabricated a sandwich structure for low hysteresis and repeatability by preventing mechanical deformation like buckling. It could be stretched by approximately 70%, and the range of the gauge factor was 2 to 14 [22]. Muth et al. attempted a new approach using carbon black and 3D printing. The mechanical reliability of their sensor was improved to eliminate interfaces that give rise to delamination between individual silicone layers. The gauge factor of the sensor was around 3.8; however, large hysteresis was observed [23]. Kong et al. made a conductive composite by dispersing carbon black nanoparticles into a PDMS matrix. This was simple to pattern in microscale onto elastomeric platforms. The developed sensor has a maximum of 10% stretchability, and its gauge factor was around 5.5 [24]. Zhou et al. developed an ultrasensitive stretchable strain sensor using a single-walled carbon nanotube (SWCNT). Its fragmented structure makes the sensor ultrasensitive to a gauge factor of 107 at a strain of 50% [25].

**Figure 1:**
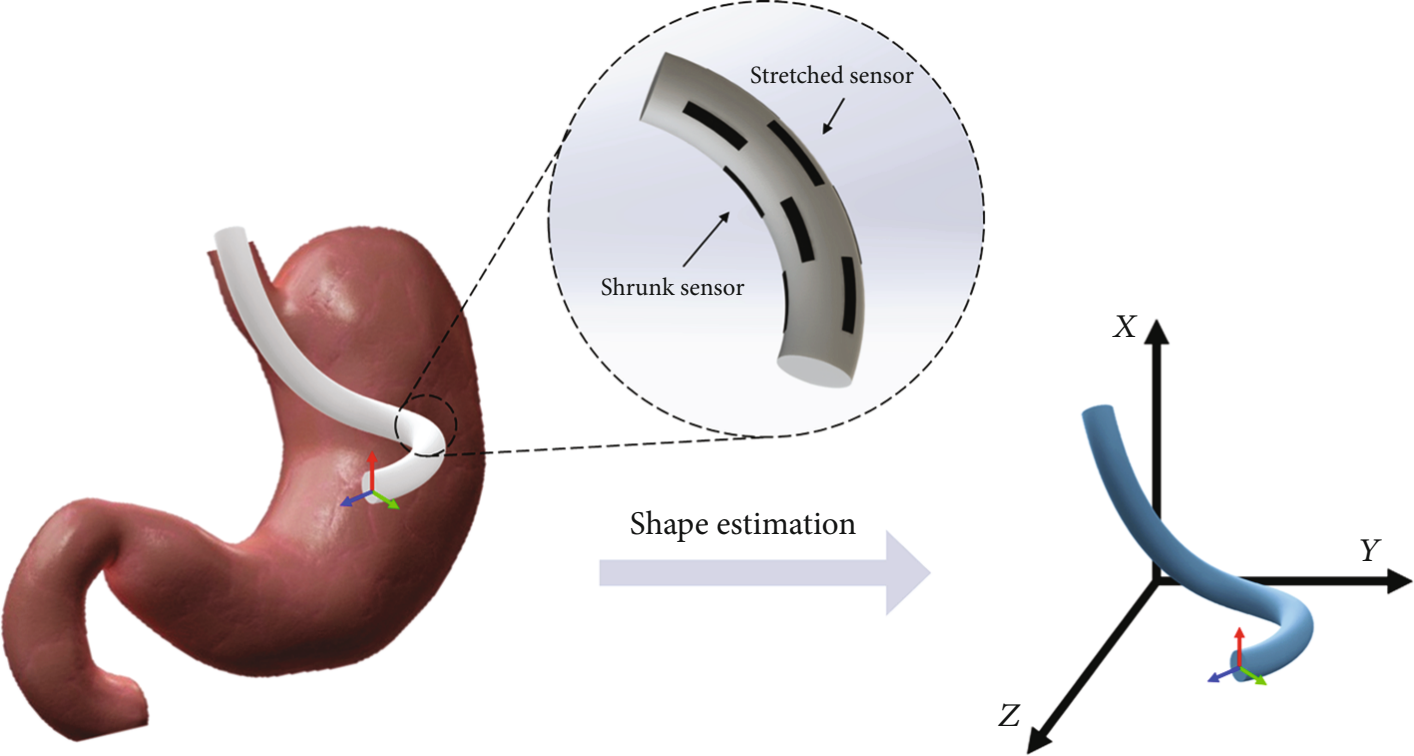
Concept design of shape estimation using stretchable strain sensor. Curvature data can be calculated from attached sensors, and the shape of the soft manipulator can be found.

In this paper, we propose a soft stretchable strain sensor composed of silicone and multiwalled carbon nanotubes (MWCNTs) for shape sensing. Sensors should be capable of deformation at least 30% to estimate both 90° of deformation of the silicone manipulator. The proposed sensor is designed as a skin-type sensor and is easy to attach to a soft manipulator without any change to the manipulator structure. Moreover, the high stretchability of the sensor makes it easy to transfer the strain manipulator to sensors. This makes it possible to estimate large deformations of the soft manipulator. The conceptual design of this idea is shown in Figure 1. Several sets of sensors are attached to the surface of the soft manipulator. The entire shape can be estimated by the attached sensor, and the user can control the manipulator more accurately. This paper is organized as follows: The sensing principle and shape reconstruction principle are discussed in Section 2. In Section 3, the design and fabrication of the sensor and the sensor-attached silicone tube are shown. Section 4 shows the experimental setup and the result of the shape estimation. Finally, a conclusion is given in Section 5.

## 2. Sensing and Shape Reconstruction Principles

### 2.1. Sensing Principle

MWCNT films have a structure in which rod-shaped MWCNTs are randomly superimposed. Electrons can move between the overlapping MWCNTs, resulting in conductivity. When strain is applied to the sensor, the distance between the MWCNTs increases, and the number of overlaps decreases. This results in reduced conductivity and increased resistance of the entire sensor. Increasingly large strains are applied, so that even if there is no overlap between the MWCNTs, they have some conductivity, which is a result of the tunnel effect. In the tunnel effect, electrons can move through the silicone substrate, and the sensors become conductive. The magnitude of the resistance showing to the tunnel effect is shown in the following equation, where $R_{\text{tunnel}}$ is the tunnel resistance; h is the Planck constant; d is the distance between CNT particles; A is the area of the cross-section; m and e are the mass and electric charge of electron, respectively; and λ is the height of the potential barrier [26]:

(1)Rtunnel=h2dAe22mλexp4πdh2mλ.

The equation shows that an increase in distance between MWCNTs leads to an exponential increase in the resistance. When the strain applied to the sensor is removed, the sensor returns to its original structure owing to the characteristics of the silicone. The distance between the distant MWCNTs decreases, and the resistance of the sensor returns to its original value. In other words, the resistance value of the sensor changes according to the distance, and the strain gauge can be fabricated using the resistance value.

### 2.2. Shape Reconstruction Principle

Moon et al. [27] used FBG sensors to measure strain. They reconstructed the entire shape of a cannula that had a 2.8 mm diameter using beam theory. Moore and Rogge [28] measured the curvature of multicore FBG sensors. They calculated the curvature using three sensors attached at equal intervals along the axis. From the calculated curvature, they reconstructed the shape using the Frenet-Serret formula. In this paper, we used the Frenet-Serret formula for a continuous curvature distribution.

#### 2.2.1. Curvature Calculation

**Figure 2:**
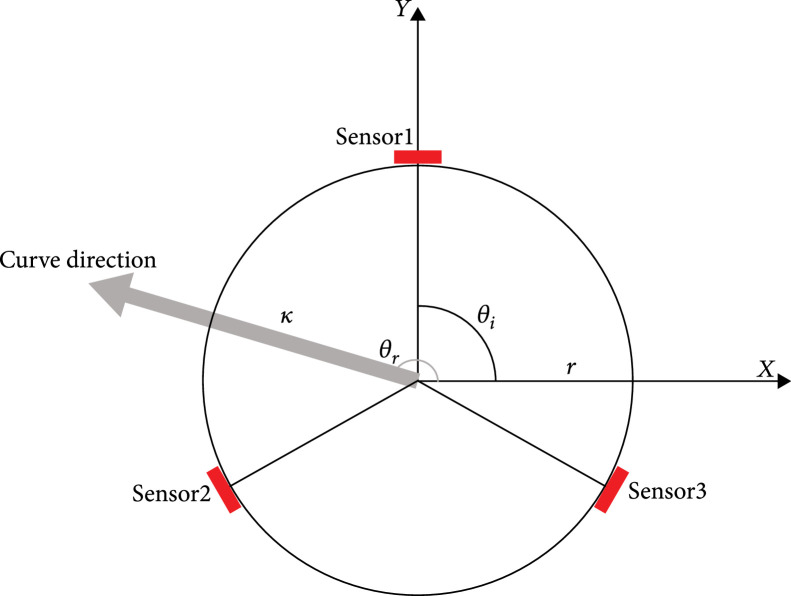
Schematic top view of silicone tube with 3 sensors. Each sensor was attached at 120° intervals. The direction of bending can be achieved from each sensor data.

The relation between the curvature and strain of three sensors was found in [28]. The relationship is as follows:

(2)κ=−∑i=13εircosθii^−∑i=13εirsinθik^,

where κ is the curvature and $\varepsilon_{i}$ and $\theta_{i}$ are the strain and location of each sensor, respectively (Figure 2). The direction of curvature can be found as follows:

(3)θk=∠κ.

#### 2.2.2. Shape Reconstruction

The Frenet-Serret formula was used to reconstruct the entire shape using the curvature. The shape of a spatial curve can be determined by the curvature $\kappa \left(s\right)$ and torsion $\tau \left(s\right)$ [29], where s is the arc length of the curve. The Frenet-Serret formula represents the relation between these two functions and the tangent unit vector $T\left(s\right)$, the normal unit vector $N\left(s\right)$, and the binormal unit vector $B\left(s\right)$:

(4)T′N′B′=0κ0−κ0τ0−τ0 TNB.

The calculated $T\left(s\right)$ can be transformed into an entire curve equation as follows:

(5)Ts=drsds,rs=∫Tsds+r0.

**Figure 3:**
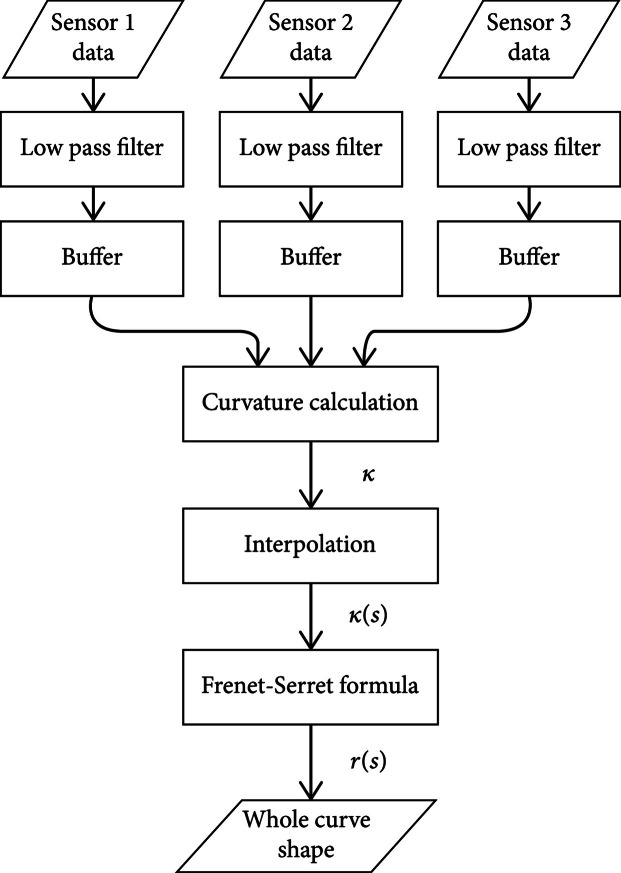
Flowchart of the whole shape estimation process.

$r\left(s\right)$ is the curve equation. The entire process is shown in Figure 3. An explanation of the low-pass filter and the buffer is in Calibration Setup.

## 3. Design and Fabrication

### 3.1. Sensor Design and Fabrication

**Figure 4:**
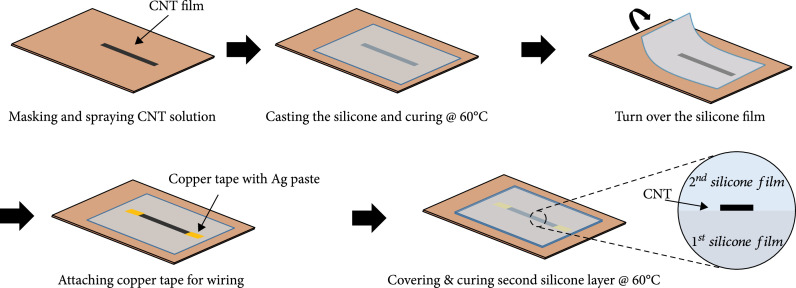
Stretchable strain sensor fabrication process with p7670 and MWCNT.

To apply the estimated shape of the soft manipulator accurately, low hysteresis and repeatability are the most important features of the sensor. If the value of the sensor is changed every measurement, the curvature value encounters errors, which creates errors for the entire shape. To prevent this, a strong binding between silicone and conductive particles is required. We used an infiltration method (refer to [22]) for strong binding. The fabrication steps are illustrated in Figure 4.

**Figure 5:**
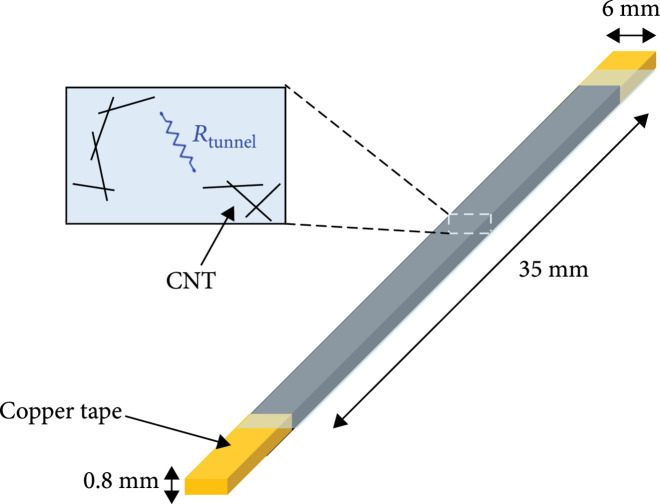
Design parameter of stretchable strain sensor for shape estimation.

First, the preparation of an MWCNT solution is needed. MWCNT was dispersed into ethanol. Here, we compared film thicknesses by spraying the same amount of MWCNT at 0.05 wt%, 0.1 wt%, and 0.5 wt% of concentration to determine which concentration makes the most porous MWCNT film. Since the same amount of MWCNT was sprayed, the thicker the film, the more porous the MWCNT film. The resulting thicknesses were 0.115 mm, 0.103 mm, and 0.102 mm. The lower the concentration of the sprayed solution, the more porous the MWCNT film. The reason is that after the ethanol evaporates, the amount of remaining MWCNT is small, and a sparse network is formed. Therefore, a 0.05 wt% concentration of solution was selected. The solution was sprayed on a teflon sheet of 80 mm×70 mm. In this step, the amount of sprayed solution determined the initial resistance of the sensors. A total of 20 ml of solution was used in this fabrication. The pattern of the MWCNT film was made using OHP film. To attach it to a silicone tube surface, the sensor size was chosen as 6 mm×35 mm (Figure 5).

After spraying, the film was heated in a 60°C oven for 20 min. Next, p7670 A and p7670 B were mixed at a ratio of 1 : 1. Silicone in a liquid state was poured on the MWCNT film, and it started to penetrate the holes in the MWCNT network. As the amount of penetration increased, the binding force became stronger. This force makes more microstructures between the MWCNTs recover from deformation when the strain is applied to the sensor and then reduced. Therefore, a porous MWCNT film is required to make a reliable and repeatable sensor. Poured silicone was flatted to a thickness of 0.4 mm and cured in a 60°C oven for 20 min.

Cured silicone layers should be turned over carefully. Then, electrodes were made to wiring using copper tape and silver paste. After that, it is covered with the same silicone and again cured in a 60°C oven for 20 min. This silicone cover protects the sensor from external damage and allows the sensor to be stable and repeatable. It prevents buckling that can occur when there is only a single layer [22]. This simple fabrication process facilitates cost-effective and fast sensor production in less than 2 hours.

### 3.2. Sensor Attachment

**Figure 6:**
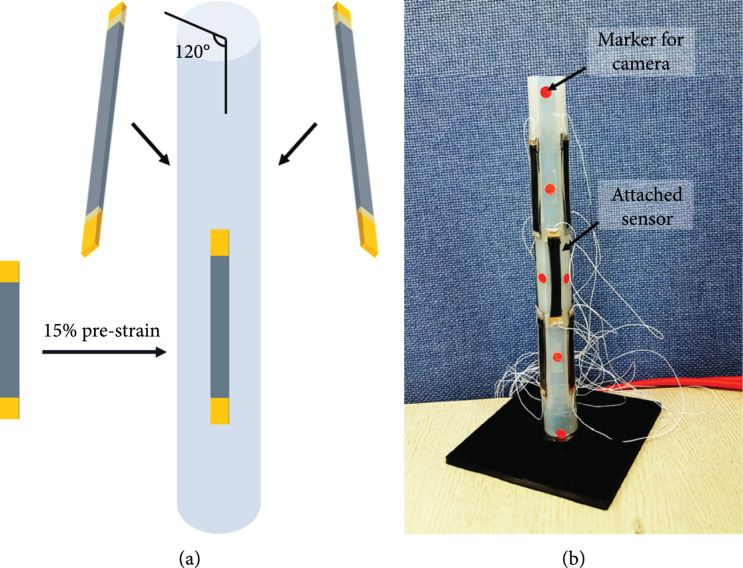
Sensor attachment method: (a) attachment of one set of sensors. One set consists of 3 sensors. Each sensor is spaced 120 degrees apart. (b) Silicone tube with 3 sets of sensors.

We used a silicone tube with a diameter of 2 cm and a length of 20 cm. We applied a 15% prestrain to the three sensors and calibrated them using the strain. This prestrain was applied to measure the negative strain when the sensor was located in the curvature direction. Three sensors were attached at 120° intervals (Figure 6).

## 4. Experiments

### 4.1. Calibration Setup

**Figure 7:**
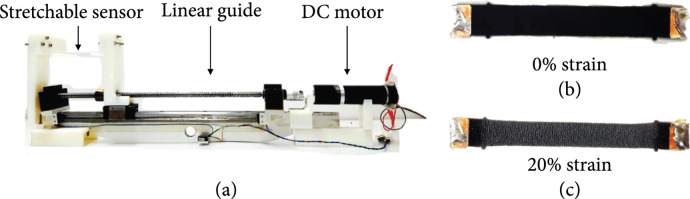
(a) Sensor calibration zig. Stretchable strain sensor with (b) 0% strain and (c) 20% strain.

To use the sensor for shape estimation, we should determine the strain from each sensor’s resistance data. For that, we calibrated a sensor using the calibration setup shown in Figure 7(a). The calibration setup consisted of a step motor, linear guide, and sensor jig. The sensor was fixed to the sensor jig, and a triangular wave and a square wave were inserted into the step motor. We observed changes in the resistance value owing to the applied strain. Data were obtained using a voltage divider circuit. To reduce noise, a low-pass filter was used. Since the resistance of the sensor was high, when the sensor was extended to its maximum length, a voltage follower buffer was used to increase the input resistance of the sensor input terminal.

### 4.2. Sensor Performance Evaluation

Before the first stretching of the sensor, the resistance was around 30 k*Ω*. There were no cracks in the sensor (Figure 7(a)), and the network of MWCNT particles was well connected. When strain was applied to the sensor, cracks began to appear. We can see the cracks in Figure 7(b). These cracks rapidly increased the total resistance of the sensor. If the strain increases, the number of cracks increases, and the size becomes uniform. After hundreds of times of applying strain and reducing it, the crack size and sensor value became stable. We called this process training. Training is essential to ensure that the resistance of the sensor is constant depending on the strain. Every experiment was conducted using the sensor after training it 500 times. The sensor cannot output the same value completely through training, but it will be able to reduce the error range between the output values during measurement. In this paper, the results of training were judged based on the last 50 data in the training process. Based on the maximum tensile resistance value, the standard deviation of the last 50 data was divided by the mean of the sensor’s resistance ($R_{\text{avg}}$):

(6)Standard deviation error=∑i=1nRi−Ravg/n−1Ravg×100%.

**Figure 8:**
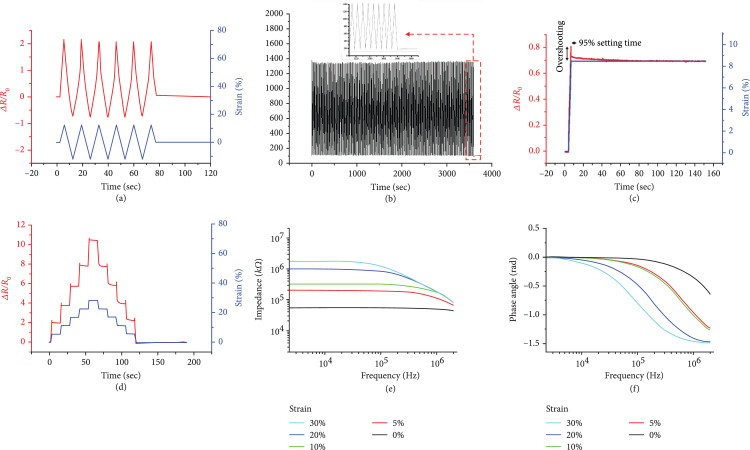
Experiment results of sensor performance: (a) response of 30% strain/release cycles; (b) response of 1000 times strain/release cycles; (c) response of single-step strain; (d) response of multistep strain; (e) frequency response of the sensor; (f) impedance and phase angle.

In Figure 8(b), the initial error was 7.6%, and it was less than 2.0% after 500 training sessions. Afterwards, when the error of all sensors is less than 2%, it is determined that training has been performed and used. As explained above, the resistance increases when strain is applied and recovers to its original value when the strain is reduced. We inserted several patterns of the strain profile to demonstrate the characteristics of the sensor.

Figure 8(a) is the result of a uniform 30% strain profile experiment. This maximum value was determined by observing the sensor resistance value under high strain. At a strain of higher than 30%, the MWCNT network was almost disconnected, and the resistance changed discretely. Therefore, the maximum stretchability to use the sensor reliably is 30%. This was sufficient to estimate the shape of the silicone tube.

The resistance was changed from an initial resistance of 97 k*Ω* to 1801 k*Ω* in the strain profile. This value can be changed depending on the amount of MWCNT solution sprayed. The resistance of the sensor was increased and decreased, following the strain profile. Under relatively low strain, we can see that the sensor value changes linearly. However, when the strain is higher than 5%, we can observe nonlinearity. The reason for this is a change in the dominant electron transfer method. As seen in equation (1), the tunnel effect exponentially increases with the distance between conductive particles. This is expressed as a nonlinear increase in the resistance of the sensor.

We can express sensitivity using a gauge factor (GF). GF is a criterion of sensitivity that expresses the characteristics of the strain gauge. The equation below shows how to obtain the GF of the sensor. R is the resistance, L is the length of the sensor, and ε is the applied strain:

(7)GF=ΔR/R0ΔL/L0=ΔR/R0ε.

Here, the GF of the sensor was 59.0. In Figure 8(a), $R_{0}$ is a graph based on the length increased by 30%. The GF calculated the resistance at maximum shrinkage as $R_{0}$. $\Delta R/R_{0}$ at this time is 11.8, and the GF is calculated as 59 for 20% strain. The GF is highly dependent on the sensing mechanism. A metal-foil-based sensor shows a range of 2–5, and a semiconductor-based sensor shows a range of 100 or larger [30]. A CNT-based sensor shows a range of 1–2.5 in [22]. The GF of our sensor is relatively high thanks to generated cracks. These cracks cause a rapid decrease in electron mobility, resulting in a high GF. A high GF increases the resolution of the shape estimation of the silicone tube.

Figure 8(b) shows the results of an experiment that extended the repetition of the strain profile to 1000 times to check the repeatability of the sensor. The maximum and minimum values were stable in the overall experiment. Repeatability is a very important characteristic of the strain sensor. Generally, a rigid sensor has better repeatability than a soft sensor. The reason is high structural deformation of the sensing part of the soft sensor owing to its intrinsic elasticity. In large deformations, it is more difficult to recover the original structure. Therefore, we used the sensor after training it hundreds of times to have a stable sensor value. It is possible to have a constant value when the position and shape of the cracks are not changed, but only the size of the cracks changes according to the strain applied. If repeatability is not guaranteed, the same sensor value will show different shape estimation results.

The elasticity of the soft sensor causes nonideal characteristics in the sensor. The overshoot behavior is one of these characteristics. We applied a step strain profile to check the overshoot behavior. Figure 8(c) indicates the step response result. We can find out small overshooting. However, it was larger when the sensor recovered from the applied strain, as shown in Figure 8(d). The reason for this is that the sensor was suddenly deformed, and the structure of the stable MWCNT changed. The overshooting value was stabilized to 95% of the final value within 100 ms after the strain was applied. This means that we can use the developed sensor in shape estimation when the estimating frequency is under 10 Hz.

Hysteresis was observed in Figure 8(d). For a strain of 0%–25%, a maximum 5.2% full-scale output of hysteresis occurred. For a strain of 25%–30%, a maximum of 10% of strain was observed. Hysteresis is a drawback of the soft sensor. This is mainly caused by the elasticity of the sensor. When a strain is applied, silicone can respond by elongating, but the MWCNT film cannot, because it is not stretchable. As a result, the MWCNT film slides between the two silicone layers. The relative position of the silicone layer and the MWCNT film is changed, which causes hysteresis. Another reason is the recovery time of the silicone substrate. It takes time for the silicone substrate to return to its original structure. During this time, the structure of the MWCNT film also changes slowly.

The tunnel effect was demonstrated by electrical impedance spectroscopy (EIS) [25]. Figures 8(e) and 8(f) show the results of EIS. When the applied strain is higher than 5%, the impedance and phase angle decrease as the frequency increases. This means the capacitance components generated by the crack are increasing in value. Electrons should tunnel through the silicone substrate between the separated MWCNT in order to be transported.

**Figure 9:**
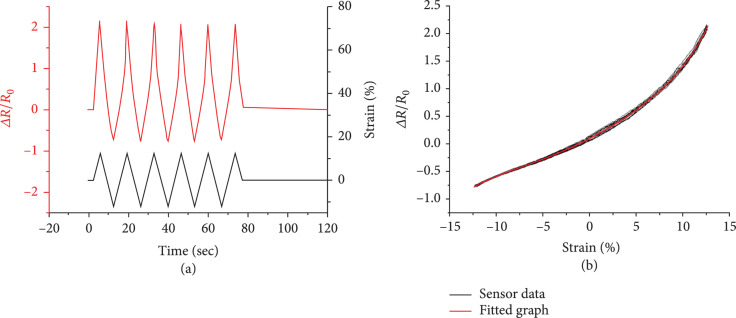
Prestrained sensor data of both sides when strain profile is applied (a). Fitted graph (b).

It is important to measure the negative strain to estimate shape. Therefore, we calibrated our system at a prestrain of 15%. Then, we inserted another 12.5% of strain on both sides (Figure 9(a)). The calibration results are shown in Figure 9(b). We use curve fitting methods to process sensor data. The fitting equation in polynomial form using the least squares method is shown below. R is the resistance, A is coefficients for polynomials, and y is fitted curve:

(8)y=A0+A1R+A2R2+A3R3+A4R4.

We can see that a larger strain creates a larger nonlinearity in Figure 9(b).

### 4.3. Shape Estimation

We strapped the thread to the end of the silicone tube with the sensor attached. Then, the actual position was determined using a 1280×720 HD camera and was compared with the estimated value. Calculating $\kappa \left(s\right)$ should be interpolated to calculate the entire shape of the curve. To compare the accuracy based on the number of sensors, experiments were conducted using three, six, and nine sensors. Sensors were attached at even intervals.

**Figure 10:**
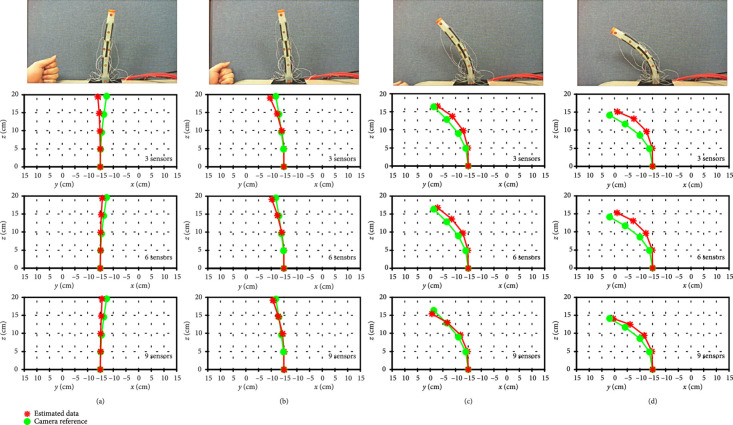
Comparison between estimated shape and camera data with various deformations (a–d).

Figure 10 shows a comparison of the results of the estimated shape and reference position data from a camera in 2D space. When the deformation of the tip position was less than 15% of the total length of the tube, the tip position error was 12.1 mm, 11.5 mm, and 8 mm using 3, 6, and 9, sensors, respectively. When the deformation was larger than 40% of the total length of the tube, the tip position error was 23 mm, 23.6 mm, and 8.9 mm, respectively. The result shows that in large deformation cases, the difference in error was relatively larger than that in small deformation cases. When the deformation was small, a small curvature was formed, and interpolation using only one point covered the entire curvature distribution without such large errors. However, when the deformation was large, the difference in curvature between sampling points became large, and the error varied greatly depending on the interpolation interval. This indicates that a tight interval of a curvature sampling point facilitates accurate shape estimation. If more sensors are attached through the miniaturization of the sensor, the error can be further reduced.

The tip position error was 4.45% for the entire length of the tube when using nine sensors. This error was caused by hysteresis of the stretchable sensor and sensor misalignment. Generally, soft sensors have larger hysteresis than rigid sensors because of large deformations. To reduce this, we need a stronger bind between conductive particles and silicone substrates for fast recovery of the microstructure when the strain is reduced. Misalignment of the sensor mounting position is another reason for errors. However, the error can be reduced by embedding the sensor by direct spraying instead of attaching the sensor to the curved surface.

To use surgical tools in clinical surgery, the tip position error should be less than 2 mm. The developed sensor was not yet suitable for clinical surgery; however, it showed the possibility of being used in the shape estimation of soft manipulators.

## 5. Conclusions

In this paper, a skin-type stretchable strain sensor for the shape estimation of a soft manipulator was developed. The stretchability of the sensor allows the sensor to be attached to the surface of the soft manipulator, making it possible to create a hollow in the center of the manipulator, and other surgical tools can be transferred through this hollow. Moreover, the simple fabrication procedure facilitates cheap and fast production.

We attached the developed sensor to a silicone tube and estimated the shape. Position error equal to 4.45% of the length of the silicone tube was observed when using nine sensors. Because of the size of the sensor, we could attach only nine sensors to the silicone tube. Three curvatures were calculated from nine sensors and interpolated to estimate the overall shape. If more sensors are attached around the silicone tube and more sensors are attached in the long run, the shape estimation will be more accurate. In future work, a method of attaching more sensors to the silicone tube surface will be studied by minimizing them. In order to minimize sensors, it is important to connect the sensor’s stretchable part and rigid electrode parts stably. Furthermore, if MWCNT can be sprayed directly on the surface of silicone tubes, errors caused by misalignment can be reduced when attaching sensors.

## Data Availability

The sensor performance and training data used to support the findings of this study have not been made available directly because the first author of the paper left the laboratory. However, to gain access to descriptions of the conditions of the experiment and how to perform it, contacting the first author of the paper is recommended.

## Supplementary Materials

Supplementary Materials This video shows the result of shape estimation using a stretchable sensor. The silicone tube has nine sensors, and three sensors measure the curvature of one position. Nine sensors are attached to the silicone tube, and the strain values of the three sensors are used to calculate the curvature of one position. The overall curvature was calculated using three curvatures to estimate the overall shape. The measured strain values of each sensor are shown on the right screen.
